# Urinary Symptoms and Sexual Dysfunction in National Level Wheelchair Rugby Male Athletes With Spinal Cord Injury

**DOI:** 10.1002/nau.70268

**Published:** 2026-03-18

**Authors:** Erica H. Gavel‐Pinos, Oluwaferanmi O. Okanlami, Michael Cottingham, Caitlin Seibel, Anne P. Cameron, Aruna V. Sarma

**Affiliations:** ^1^ Faculty of Kinesiology, Sport, and Recreation University of Alberta Edmonton Alberta Canada; ^2^ Department of Urology University of Michigan Ann Arbor Michigan USA; ^3^ Health and Human Performance University of Houston Houston Texas USA

**Keywords:** bladder dysfunction, parasport, quality of life, sexual function, spinal cord injury, wheelchair rugby

## Abstract

**Background:**

Spinal cord injury (SCI) results in a disruption of autonomic function negatively affecting bladder and sexual function. Research suggests that sport participation can improve physiological function and quality of relationships. This study aimed to describe the prevalence of bladder and sexual dysfunction in national level wheelchair rugby (WCR) males with a SCI.

**Methods:**

Sixty‐nine male WCR athletes completed a self‐reported questionnaire during national competition. Participants provided information on sociodemographic, injury and sport characteristics, and completed the Neurogenic Bladder Symptom Score Short Form (NBSS‐SF) and the International Index of Erectile Function (IIEF).

**Results:**

The NBSS‐SF depicted an overall score of 9.8 ± 4.2, 3.2 ± 2.4 in incontinence, 4.1 ± 2.0 for storage and voiding, and 2.5 ± 1.2 in the consequence domains. Athletes with > 10‐years of experience had significantly better bladder function, compared to those with < 10‐years (*p* = 0.036). The IIEF showed values of 19.3 ± 7.0 for erectile function, 4.6 ± 2.4 in orgasmic function, 8.0 ± 1.8 in sexual desire, 11.8 ± 3.6 for intercourse satisfaction, and 7.7 ± 2.1 for overall satisfaction. Athletes with < 2‐years of experience had notably lower scores in some domains.

**Conclusions:**

While injury classification and completeness did not show differences in functional scores, long‐term WCR participation was associated with significantly better bladder function, suggesting a rehabilitative benefit of sustained sport engagement.

## 1 Introduction

According to the World Health Organization, an estimated 250 000–500 000 people sustain a spinal cord injury (SCI) annually, with the largest demographic being males aged 20–29 years (WHO 2023). Although the completeness and level of SCIs vary, a considerable number of males—particularly those with a cervical SCI (cSCI)—experience erectile dysfunction (ED) and neurogenic lower urinary tract dysfunction (NGLUTD) with resulting bladder dysfunction due to significant neurophysiological impairment [1, 2].

ED is defined as the inability to achieve or maintain an erection sufficient for satisfactory sexual intercourse [2], whereas NGLUTD refers to abnormal function of either the bladder, bladder neck, and/or its sphincters related to a neurologic disorder [3]. The prevalence and severity of ED and bladder dysfunction is largely dependent on the injury location and completeness, with individuals sustaining injuries at the fifth lumbar vertebra or higher being highly likely to experience some degree of ED and bladder dysfunction [4, 5] Consequently, infertility and sexual dysfunction are major concerns in this population with estimates suggesting that approximately 90% of men with SCI are unable to conceive naturally [6, 7]. Moreover, bladder dysfunction of sufficient severity to require bladder catheterization occurs in over 80% of individuals with SCI [8] and has been shown to alter relationships, impact the involvement in social activities, and contribute to challenges such as anxiety and urinary tract infections [9]. Sport, characterized as physical activity involving exertion and hand‐eye coordination, commonly occurs within social contexts and spans from recreational to elite levels. The three primary domains of sport are physical, psychological, and social [10, 11]. Given the physical and social nature of sport, especially wheelchair sports, it represents a valuable setting for peer‐to‐peer mentoring and rehabilitation, particularly for individuals with acquired cSCI [12]. However, data exploring the relationships among sexual health, bladder and urinary dysfunction, wheelchair sport participation, and cSCI remain limited and understudied. Therefore, the primary aim of this study is to assess sexual health and bladder and urinary dysfunction in male athletes with cervical spinal cord injury.

## 2 Materials and Methods

### 2.1 Population and Setting

Sixty‐nine (*n* = 69) national‐level wheelchair rugby (WCR) athletes volunteered to participate in this study (Table 1). Data were collected via surveys administered at the 2023 National Championship held in Rockford, Illinois, over a 3‐day period. Eligible participants were athletes aged 18 years or older with a spinal cord injury (SCI) at the T6 level or above. Exclusion criteria included having an SCI below T6, lack of consent, incomplete questionnaire responses, age under 18, or participation at the international competition level. Prior to data collection, all participants provided written and oral informed consent after being fully informed of the study protocol and their rights.

**Table 1:** Descriptive and clinical characteristics of the male participants (*n* = 69).

Characteristics	N (%)
**Age**	
< 20	1 (1.5%)
21−30	14 (20%)
31–40	23 (33%)
41–50	20 (29%)
> 51	11 (16%)
**Race/Ethnicity**	
White	57 (83%)
African American	7 (10%)
American Indian or Alaska Native	2 (2.9%)
Other	3 (4.4%)
**Level of injury**	
C5 complete	4 (5.8%)
C5 incomplete	14 (20%)
C6 complete	11 (16%)
C6 incomplete	21 (30%)
C7 complete	5 (7.3%)
C7 incomplete	6 (8.7%)
Other	8 (12%)
**Classification**	
0.5–1.0	23 (33%)
1.5–2.0	27 (39%)
2.5–3.0	17 (25%)
3.5	2 (2.9%)
**Years involved with WCR**	
0–2	3 (4.4%)
3–5	11 (16%)
6−10	21 (30%)
> 10	34 (49%)

### 2.2 Data Collection

The research instrument was an anonymous questionnaire completed by athletes either prior to or during competition. The survey assessed demographics, SCI characteristics, WCR experience, sexual health, and bladder function. Many items were adapted from validated instruments including the International Index of Erectile Function (IIEF) [13] and the Neurogenic Bladder Symptom Score Short Form (NBSS‐SF) [14] described below, supplemented with additional questions tailored to this population. The survey was administered via Qualtrics software (XM, Qualtrics, Provo, Utah, USA) on iPads (10th generation, Apple, Cupertino, California, USA) and designed to be completed within 15 min.

The IIEF is a widely used, validated, and reliable instrument for assessing male sexual health, particularly among individuals with spinal cord injury (SCI). The questionnaire includes 15 items distributed across five domains (score ranges): erectile function (0–30), orgasmic function (0–10), sexual desire (1–10), intercourse satisfaction (0–15), and overall satisfaction (1–10) [13]. Each item is scored on a Likert scale, and the total score reflects the participant's sexual functioning. A cumulative score of 25 or lower is indicative of clinically significant erectile dysfunction. The IIEF is considered sensitive for diagnosing conditions related to arousal, desire, and sexual pain disorders.

The Neurogenic Bladder Symptom Score – Short Form (NBSS‐SF) is a validated tool designed to evaluate the burden of bladder‐related symptoms in individuals with neurogenic bladder dysfunction [14]. The instrument comprises three domains—storage and voiding (0–9), incontinence (0–12), and consequence (0–7)—with a total score ranging from 0 (minimal symptom burden) to 28 (maximum symptom burden). Higher scores indicate greater severity of symptoms and overall dysfunction. The NBSS‐SF provides a concise yet comprehensive measure of neurogenic bladder symptomatology in clinical and research settings.

### 2.3 Statistical Analysis

Descriptive statistics were used to summarize participant demographics and clinical characteristics. Continuous variables, including scores from the NBSS‐SF and IIEF, were reported as means with standard deviations. Categorical variables, such as age group, race/ethnicity, and injury classification, were presented as frequencies and percentages. Group comparisons of NBSS‐SF and IIEF domain scores were conducted across several independent variables, including SCI classification level (0.5–1.0, 1.5–2.0, 2.5–3.5), completeness of spinal cord injury (complete vs. incomplete), and years of involvement in wheelchair rugby (0–2, 3–5, 6–10, > 10 years). The Kruskal‐Wallis test was used to assess differences across multiple groups, while Wilcoxon rank tests were used for binary group comparisons. A p‐value of < 0.05 was considered statistically significant. All analyses were conducted using SAS 9.4 statistical software (SAS Institute, Cary, North Carolina, USA).

## 3 Results

The study included 69 male national‐level wheelchair rugby athletes with SCI. The majority were aged 31–50 years (62%), and most participants identified as White (83%), followed by African American (10%) and other racial/ethnic groups. The most common levels of injury were C6 incomplete (30%) and C5 incomplete (20%). The most frequent SCI classification was 1.5–2.0 (39%), and nearly half (49%) had participated in wheelchair rugby for over 10 years (Table 1).

**Figure 1:**
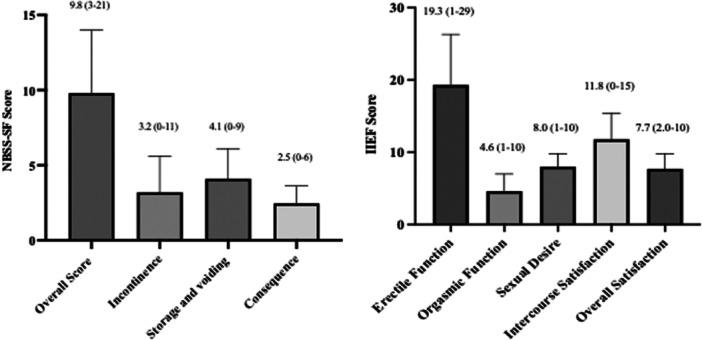
Overall mean Neurogenic Bladder Symptom Score Short‐Form (NBSS‐S) and International Index for Erectile Function (IIEF) scores for national‐level wheelchair rugby (WCR) athletes. Scores are presented as mean (range) above each bar.

Mean (±SD) NBSS‐SF overall score was 9.8 (±4.2), with subdomain means of 3.2 (±2.4) for incontinence, 4.1 (±2.0) for storage/voiding, and 2.5 ( ± 1.2) for consequences. The mean (±SD) IIEF overall score was 52.0 (±13.7), with erectile function averaging 19.3 (±7.0), orgasmic function 4.6 (±2.4), sexual desire 8.0 (±1.8), intercourse satisfaction 11.8 (±3.6), and overall satisfaction 7.7 (±2.1) (Figure 1).

When stratified by SCI classification (Table 2), NBSS‐SF scores did not significantly differ across classification levels. IIEF erectile function scores trended lower in Class 1.5–2.0 compared to the other groups (17.0 vs. 20.5 and 21.2) (*p* = 0.06). Similarly, overall IIEF scores were lowest in Class 1.5–2.0 (*p* = 0.08). Comparison by injury completeness (Table 3) showed no significant differences in NBSS‐SF scores. Participants with incomplete injuries reported higher erectile function scores than those with complete injuries (20.0 vs. 16.2) (*p* = 0.08). Other IIEF domains were not significantly different between groups.

**Table 2:** Bladder and sexual function scores by SCI classification.

	Class 0.5–1.0 (*n* = 23)	Class 1.5–2.0 (*n* = 27)	Class 2.5–3.5 (*n* = 19)	*p* value*
**NBSS‐SF Domains** (mean ± sd)				
Overall score	9.7 ± 4.1	10.4 ± 4.6	9.1 ± 3.9	0.6
Incontinence	3.4 ± 2.2	3.2 ± 2.6	3.1 ± 2.6	0.9
Storage and voiding	3.8 ± 2.1	4.5 ± 2.0	3.7 ± 1.5	0.3
Consequence	2.5 ± 1.0	2.7 ± 1.3	2.2 ± 1.3	0.5
**IIEF Domains** (mean ± sd)				
Overall score	52.3 ± 15.3	47.6 ± 12.2	55.7 ± 11.0	0.08
Erectile Function	20.5 ± 7.9	17.0 ± 6.7	21.2 ± 5.5	0.06
Orgasmic Function	4.7 ± 2.4	4.0 ± 2.1	5.3 ± 2.7	0.3
Sexual Desire	8.0 ± 2.4	7.9 ± 1.7	8.2 ± 1	0.8
Intercourse Satisfaction	11.7 ± 4.2	11.3 ± 3.7	12.6 ± 2.6	0.4
Overall Satisfaction	7.5 ± 1.9	7.3 ± 2.3	8.4 ± 1.9	0.2

* *p* value based on Kruskal‐Wallis test.

**Table 3:** Bladder and sexual function scores by complete versus incomplete SCI status.

	Complete (*n* = 20)	Incomplete (*n* = 41)	*p* value*
**NBSS‐SF Domain** (mean ± sd)			
Overall score	9.7 ± 4.2	9.7 ± 4.5	> 0.9
Incontinence	3.0 ± 2.5	3.3 ± 2.6	0.6
Storage and voiding	4.0 ± 2.4	4.1 ± 1.8	0.7
Consequence	2.8 ± 1.0	2.4 ± 1.3	0.1
**IIEF Domain** (mean ± sd)			
Overall score	46.8 ± 15.0	52.3 ± 12.6	0.2
Erectile Function	16.2 ± 8.1	20.0 ± 6.4	0.08
Orgasmic Function	3.9 ± 2.3	4.7 ± 2.3	0.13
Sexual Desire	8.2 ± 1.6	8.0 ± 1.6	0.8
Intercourse Satisfaction	11.1 ± 4.4	12.0 ± 3.4	0.5
Overall Satisfaction	7.5 ± 2.1	7.6 ± 2.2	0.7

* *p* value based on Wilcoxon rank test.

Participants involved in wheelchair rugby for more than 10 years reported significantly lower NBSS‐SF overall (8.8 ± 4.7) and incontinence scores (2.6 ± 2.8) compared to those with shorter participation durations (*p* = 0.04 and *p* = 0.02, respectively) (Table 4). While IIEF scores were generally higher with increasing years of participation, findings did not statistical significance. Notably, those in the shortest duration group (0–2 years) had the lowest IIEF scores across all domains.

**Table 4:** Bladder and sexual function scores by number of years involved in WCR.

	0–2 years (*n* = 3)	3–5 years (*n* = 11)	6–10 years (*n* = 21)	> 10 years (*n* = 34)	*p* value*
**NBSS‐SF Domain** (mean ± sd)					
Overall score	10.7 ± 3.5	11.5 ± 4.4	10.5 ± 3.1	8.8 ± 4.7	0.04
Incontinence	3.7 ± 0.6	3.9 ± 2.8	3.9 ± 1.5	2.6 ± 2.8	0.02
Storage and voiding	4.0 ± 1.7	4.5 ± 1.8	4.4 ± 2.1	3.8 ± 2.0	0.6
Consequence	3.0 ± 1.7	3.1 ± 1.1	2.2 ± 1.2	2.4 ± 1.2	0.3
**IIEF Domain** (mean ± sd)					
Overall score	34.3 ± 20.5	49.0 ± 17.8	54.6 ± 9.5	51.7 ± 12.3	0.4
Erectile Function	10.7 ± 7.2	18.6 ± 8.5	20.5 ± 5.7	19.6 ± 7.0	0.2
Orgasmic Function	3.0 ± 1.7	4.5 ± 2.5	5.3 ± 2.6	4.3 ± 2.3	0.3
Sexual Desire	9.3 ± 1.2	7.4 ± 2.5	8.2 ± 2.0	8.0 ± 1.5	0.4
Intercourse Satisfaction	6.3 ± 7.8	10.7 ± 4.6	12.5 ± 1.7	12.2 ± 3.3	0.5
Overall Satisfaction	5.0 ± 3.5	7.8 ± 1.7	8.1 ± 1.9	7.6 ± 2.1	0.4

* *p* value based on Kruskal‐Wallis test.

## 4 Discussion

This study examined bladder and sexual function among national‐level male wheelchair rugby (WCR) athletes with cervical spinal cord injuries (cSCI), with particular attention to SCI classification, SCI completeness, and duration of WCR involvement. Our findings suggest that while bladder and sexual dysfunction are common in this population, longer‐term engagement in WCR may be associated with improved outcomes.

The current study reported moderate scores for overall neurogenic bladder symptoms, with no significant difference between classification or completeness. Although this is the only study to analyze NBSS‐SF in athletes with a cSCI, these results are relatively lower (better function) to those reported by Welk and Carlson [14] and Welk and Lenherr [15] using the original NBSS form. The studies by Welk and Carlson [14] and Welk and Lenherr [15] included an array of participants who either had a SCI, multiple sclerosis, or congenital neurogenic bladder without any between‐group comparisons making it difficult to compare. However, one could suggest that given the present population may be health focused given their participation in elite sports, have good medical care and strong social supports, WCR could facilitate improved NBSS scores independent of disability, classification, or type of scale used.

Although there were no differences between classification or completeness, the most notable finding was the significantly lower bladder symptom burden among athletes with more than 10 years of WCR participation. These individuals reported significantly better overall NBSS‐SF scores (*p* = 0.001), with particularly lower incontinence subdomain scores (*p* = 0.001). While causality cannot be inferred in this cross‐sectional study, the association suggests a potential protective or adaptive effect of long‐term sport participation on bladder health. Physical activity has been associated with improved autonomic regulation and urological function in other populations with SCI [12], and the social and rehabilitative aspects of WCR may facilitate better symptom awareness, management strategies, and access to peer support.

Sexual health scores did not significantly differ by years of WCR involvement, though athletes with < 2 years of experience had notably lower scores in some domains relative to those with longer WCR participation. Erectile function trended lower in those with complete injuries, aligning with prior studies indicating that more severe injury is associated with greater disruption to sexual health [4, 7]. Despite the observed trends, no significant differences in sexual function were found across years of WCR involvement. While previous research has suggested that physical activity can improve sexual health and psychosocial outcomes, it is possible that neurophysiological limitations, stigma, and lack of targeted sexual health interventions in this population may limit measurable improvements in sexual function over time [5].

### 4.1 Clinical Implications

The current study supports pre‐existing literature in the areas of sexual health and bladder dysfunction in people with a SCI. From a clinical perspective, the results of this study suggest that team sport may help support clinical recommendations from both a sexual health and bladder perspective. However, the impact of WCR in sexual health and bladder dysfunction may impact one on different timelines. Although this study contributes to the literature in athletes with a SCI and the impact on sexual health and bladder dysfunction, additional research is needed in this particular area.

### 4.2 Limitations

This study is limited by its cross‐sectional design and self‐reported measures, which may be subject to recall or social desirability bias. While the study was additionally limited in its sample size, this is the largest known study of athletes with a SCI to date. Further, the sample was restricted to national‐level athletes lacking a control population, limiting generalizability to less active or non‐athlete SCI populations. Finally, the absence of neurophysiological data on injury severity and urodynamic assessments limits the ability to fully interpret functional outcomes.

## 5 Conclusions

This study highlights the ongoing challenges related to bladder and sexual dysfunction among male wheelchair rugby athletes with cervical spinal cord injuries which remain highly prevalent. However, findings suggest longer participation in adaptive sport may be associated with modest improvements in symptom burden, highlighting the potential rehabilitative benefits of sustained physical activity. Our findings underscore the importance of incorporating sexual and bladder health into routine care and rehabilitation for men with cSCI, including those involved in sport.

## Ethics Statement

This study was conducted in accordance with the Declaration of Helsinki and approved by the University of Michigan Institutional Review Board. All participants provided written informed consent prior to enrollment.

## Consent

Written informed consent was obtained from all participants prior to study enrollment. Permission to reproduce any previously published material (including figures, tables, or text excerpts) has been obtained from the copyright holders. Appropriate credit has been given in the figure legends and reference list in accordance with journal guidelines.

## Conflicts of Interest

The authors declare no conflicts of interest.

## Data Availability

The data that support the findings of this study are available from the corresponding author upon reasonable request.

## References

[1] R. Hamid , M. A. Averbeck , H. Chiang , et al., “Epidemiology and Pathophysiology of Neurogenic Bladder After Spinal Cord Injury,” World Journal of Urology 36, no. 10 (2018): 1517–1527, 10.1007/s00345-018-2301-z. 29752515

[2] F. A. Yafi , L. Jenkins , M. Albersen , et al., “Erectile Dysfunction,” Nature Reviews Disease Primers 2 (2016): 16003, 10.1038/nrdp.2016.3. PMC5027992 27188339

[3] D. A. Ginsberg , T. B. Boone , A. P. Cameron , et al., “The AUA/SUFU Guideline on Adult Neurogenic Lower Urinary Tract Dysfunction: Diagnosis and Evaluation,” Journal of Urology 206, no. 5 (2021): 1097–1105, 10.1097/ju.0000000000002235. 34495687

[4] A. Krassioukov , “Autonomic Function Following Cervical Spinal Cord Injury,” Respiratory Physiology & Neurobiology 169, no. 2 (2009): 157–164, 10.1016/j.resp.2009.08.003. 19682607

[5] I. S. de Tejada , I. Goldstein , K. Azadzoi , R. J. Krane , and R. A. Cohen , “Impaired Neurogenic and Endothelium‐Mediated Relaxation of Penile Smooth Muscle From Diabetic Men With Impotence,” New England Journal of Medicine 320, no. 16 (1989): 1025–1030, 10.1056/nejm198904203201601. 2927481

[6] L. L. Chapelain , P. N. Van Tam , P. Dehail , et al., “Ejaculatory Stimulation, Quality of Semen and Reproductive Aspects in Spinal Cord Injured Men,” Spinal Cord 36, no. 2 (1998): 132–136, 10.1038/sj.sc.3100482. 9495004

[7] E. Ibrahim , C. M. Lynne , and N. L. Brackett , “Male Fertility Following Spinal Cord Injury: An Update,” Andrology 4, no. 1 (2016): 13–26, 10.1111/andr.12119. 26536656

[8] A. P. Cameron , L. P. Wallner , D. G. Tate , A. V. Sarma , G. M. Rodriguez , and J. Q. Clemens , “Bladder Management After Spinal Cord Injury in the United States 1972 to 2005,” Journal of Urology 184, no. 1 (2010): 213–217, 10.1016/j.juro.2010.03.008. 20478597

[9] S. Braaf , A. Lennox , A. Nunn , and B. Gabbe , “Social Activity and Relationship Changes Experienced by People With Bowel and Bladder Dysfunction Following Spinal Cord Injury,” Spinal Cord 55, no. 7 (2017): 679–686, 10.1038/sc.2017.19. 28244500

[10] R. M. Eime , J. A. Young , J. T. Harvey , M. J. Charity , and W. R. Payne , “A Systematic Review of the Psychological and Social Benefits of Participation in Sport for Adults: Informing Development of a Conceptual Model of Health Through Sport,” International Journal of Behavioral Nutrition and Physical Activity 10, no. 1 (2013): 135, 10.1186/1479-5868-10-135. 24313992 PMC4028858

[11] L. Cheung , K. Chan , M. G. Heffernan , et al., “The Impact of Sport Participation for Individuals With Spinal Cord Injury: A Scoping Review,” Neurorehabilitation 51, no. 3 (2022): 353–395, 10.3233/NRE-220037. 36057799

[12] R. C. Rosen , A. Riley , G. Wagner , I. H. Osterloh , J. Kirkpatrick , and A. Mishra , “The International Index of Erectile Function (IIEF): A Multidimensional Scale for Assessment of Erectile Dysfunction,” Urology 49, no. 6 (1997): 822–830, 10.1016/s0090-4295(97)00238-0. 9187685

[13] B. Welk , S. Lenherr , S. Elliott , et al., “The Creation and Validation of a Short Form of the Neurogenic Bladder Symptom Score,” Neurourology and Urodynamics 39, no. 4 (2020): 1162–1169, 10.1002/nau.24336. 32196732

[14] B. Welk , K. Carlson , and R. Baverstock , “A Pilot Study of the Responsiveness of the Neurogenic Bladder Symptom Score (NBSS),” Canadian Urological Association Journal 11, no. 12 (2017): 376–378, 10.5489/cuaj.4833. 29257742 PMC5962943

[15] B. Welk , S. Lenherr , S. Elliott , et al., “The Neurogenic Bladder Symptom Score (NBSS): A Secondary Assessment of Its Validity, Reliability Among People With a Spinal Cord Injury,” Spinal Cord 56, no. 3 (2018): 259–264, 10.1038/s41393-017-0028-0. 29184133

